# Intrauterine autologous platelet-rich plasma treatment in women with at least two implantation failures: A retrospective cohort study

**DOI:** 10.18502/ijrm.v22i1.15236

**Published:** 2024-02-23

**Authors:** Neda Fattahi Meybodi, Maryam Eftekhar, Behnaz Gandom

**Affiliations:** Research and Clinical Center for Infertility, Yazd Reproductive Sciences Institute, Shahid Sadoughi University of Medical Sciences, Yazd, Iran.

**Keywords:** Platelet-rich plasma, Embryo implantation, Assisted reproductive technology, Ovum donor, Live birth rate.

## Abstract

**Background:**

Finding the most effective way to improve implantation rate in women who are receiving assisted reproductive technology treatment is still a challenge.

**Objective:**

This study aimed to assess the pregnancy outcomes of intrauterine platelet-rich plasma (PRP) therapy in women with a history of at least 2 implantation failures.

**Materials and Methods:**

In this retrospective cohort study, data of 852 women who were candidates for frozen-thawed embryo transfer was extracted from their medical records from April 2017 to September 2021 at Yazd Reproductive Sciences Institute, Yazd, Iran. Of these, 432 received intrauterine PRP treatment 48 hr before transfer (PRP group), and the results of the pregnancy outcomes compared with 420 of the control group who did not receive the treatment before transfer.

**Results:**

Pregnancy outcomes, including chemical, clinical, ongoing pregnancy, and live birth rate were statistically significant in the PRP group (p < 0.001). However, when categorized according to the implantation history, this significant improvement in all 4 was only seen in women with at least 2 prior implantation failures. In women with a history of only one implantation failure, PRP therapy significantly improved the ongoing pregnancy and live birth rate (19.5%, p = 0.04). Also, in women who received donor eggs and had repeated implantation failure, PRP improved pregnancy outcomes clinically but not statistically (p = 0.15).

**Conclusion:**

PRP seems to be effective in improving the pregnancy rate in women with a history of 2 or more implantation failures and also shows an increase in the live birth rate in women with only one implantation failure.

## 1. Introduction

Implantation is a sophisticated matter that needs both a good quality embryo and a receptive and prepared endometrium to work in harmony. The implantation process consists of 3 stages: apposition, adhesion, or invasion (1-3).

A successful implantation is considered a visualization of the pregnancy sac at the 5 ${\;}^{\text{th}}$ wk. On ultrasound after transferring the embryo, the factors that are involved in the process can be categorized into 4 main groups: embryonic quality; embryo-endometrial cross-talk; the regulation of maternal immunologic mediators; and endometrial receptivity. If any of these face difficulties despite the presence of a good-quality embryo, implantation failure might occur (4-6).

Several methods have been considered for improving the implantation rate in assisted reproductive technology (ART), but the most effective one remains unclear. Intrauterine infusion of peripheral blood mononuclear cells, growth hormone, and granulosa colony-stimulating factor are just a few examples.

Tubal ligation and salpingectomy for the treatment of hydrosalpinx, assessing the uterine cavity with hysteroscopy, and performing endometrial scratching are other options. In the lab environment, transferring the embryo in the blastocyst stage and performing preimplantation genetic screening on embryos before the transfer also have been considered (7-10).

It has been shown that endometrial mesenchymal stem cells and endometrial stromal fibroblasts as well as other cellular components have a key role in blastocyst implantation and promote cell adhesion and immunological responses, it seems PRP enhances their movement during the process and therefore improve the chance of pregnancy. On the other hand, PRP also has stimulatory effects on the function and production of various growth factors that are involved in cell attraction, migration, and transformation as well as vascularization and inflammation, which all are crucial steps in a successful implantation (11).

Intrauterine infusion of PRP as a rich source of several cytokines and growth factors, including insulin-like growth factor I, II, fibroblast growth factor, and interleukin 8, just to name a few, may affect the process of growth in the endometrium and its receptiveness to the embryo (12-15).

This study aimed to assess the effectiveness of intrauterine infusion of PRP on pregnancy outcomes in women with a history of at least 2 implantation failures.

## 2. Materials and Methods

In this retrospective cohort study, data from 852 frozen-thawed embryo transfer (FET) candidates aged between 18 and 42 yr was extracted from their medical records from April 2017 to September 2021 in Yazd Reproductive Sciences Institute, Yazd, Iran.

Women with known uterine anomalies (congenital or acquired), thrombophilia, and uncontrolled endocrine disorders such as hypothyroidism and hyperprolactinemia were excluded.

To prepare the endometrium for transfer, both the control (n = 420) and PRP group (n = 432) received 6 mg/day of estradiol valerate from the second day of menstruation and on the 13 ${\;}^{\text{th}}$ day of menstrual cycle vaginal sonography was performed. If the endometrial thickness was at least ≥ 7 mm then 400 mg vaginal progesterone was prescribed every 12 hr for 3 days.

In the PRP group 2 days before transfer, 8.5 ml of blood was taken into an anticoagulated syringe with anticoagulant citrate dextrose-A solution and then centrifuged at 1600 g for 10 min. The plasma layer and buffy coat were transferred into another syringe and centrifuged again at 3500 g for 5 min. The final product would then be 1.5 ml PRP with 4-5 times platelet concentration more than peripheral blood. Then 0.5-1 cc of PRP was administered intrauterine (14).

All women returned for embryo transfer after 3 days of progesterone administration. Estradiol tablets and vaginal progesterone were continued until the 10 ${\;}^{\text{th}}$ wk of pregnancy.

On the 14 ${\;}^{\text{th}}$ day of embryo transfer, the level of beta human chorionic gonadotropin hormone was measured, and any level above 50 IU/L was considered a positive chemical pregnancy. Detection of fetal heart activity 2 wk after a positive beta-human chorionic gonadotropin hormone confirmed a clinical pregnancy. Ongoing pregnancy was defined as an established pregnancy after the 12 ${\;}^{\text{th}}$ wk of gestation. The live birth rate was considered the birth of a live fetus after 28 gestational weeks.

Factors such as age, type of ART cycle, number, and quality of retrieved oocytes and embryos were analyzed.

### Sample size

A total of 432 women received PRP treatment 48 hr before transfer. To ensure a suitable power for the study, we allocated a 1:1 ratio for the control group. A total of 3351 cases of FETs were matched in our inclusion and exclusion criteria in this period. From this, we chose the first of every 8 files, using systematic sampling and 420 women files enrolled in the control group.

From 432 women in PRP group, 217 cases had a history of at least 2 prior implantation failures, and from 420 women in control group 101 had a history of 2 or more implantation failures.

### Ethical considerations

The study protocol was approved by the Ethics Committee of Yazd Research and Clinical Center for Infertility, Yazd, Iran (Code: IR.SSU.RSI.REC.1401.006).

### Statistical analysis

Data were analyzed using the SPSS software (Statistical Package for Social Sciences, SPSS Inc version 2.0., Chicago, Illinois, USA). The Chi-square, Mann-Whitney, and student's *t* tests were used to evaluate the relation between variables. P-value < 0.05 was considered statistically significant.

## 3. Results

In this study, data from 852 women were analyzed, with 432 in the PRP group and 420 in the control group. Of these, 318 had a history of 2 or more implantation failures, with 217 in the PRP group and 101 in the control group.

Demographic characteristics between the PRP and control groups showed no significant differences, irrespective of implantation failure history (Tables I and II). Overall pregnancy outcomes, including chemical, clinical, ongoing pregnancy rate, and live birth rate, significantly improved in the PRP group (Table III).

However, when considering the history of implantation failure, improvements in all 4 categories were evident only in women with at least 2 prior failures. For those with a history of one implantation failure, PRP significantly improved ongoing pregnancy and live birth rates, but chemical and clinical pregnancy rates lacked statistical significance (Table IV).

The study also assessed outcomes for 269 women who received donor eggs, with 165 undergoing PRP treatment before transfer. Chemical and clinical pregnancy rates showed statistical improvement in the PRP group. Although ongoing pregnancy and live birth rates were higher, the results were not statistically significant (Table V).

**Table 1:** Demographic characteristics of participants in 2 study groups


**Variables**		**PRP group (n = 432)**	**Control group (n = 420)**	**P-value**
Age (yr)*		34.31 ± 5.95 (7, 31)	33.66 ± 6.10 (7, 31)	0.11
BMI (kg/m^2^)*		26.42 ± 4.19 (4.6, 26)	26.47 ± 4.28 (5.1, 25)	0.86
Duration of infertility (year)**		7.07 ± 4.66 (6.5, 6.00)	6.67 ± 4.40 (6.0, 5.50)	0.24
COC**		18.13 ± 9.52 (12, 17)	18.81 ± 9.48 (13, 18)	0.13
Total number of embryos**		9.65 ± 5.96 (8, 8)	9.20 ± 5.01 (6, 8)	0.82
Number of transferred embryo**		1.92 ± 0.31 (0, 2)	1.95 ± 0.25 (0, 2)	0.21
Embryo quality***				
	**Good (A/B)**	405 (93.8)	403 (96)	
	**Bad (C/D)**	27 (6.2)	17 (4)	0.16
Type of fertilization***				
	**IVF**	27 (6.2)	34 (8.1)	
	**ICSI**	218 (50.5)	222 (52.9)	
	**IVF+ICSI**	187 (43.3)	164 (39)	0.33
ART cycle***				
	**Antagonist**	411 (95.1)	408 (97.1)		
	**Agonist**	16 (3.7)	7 (1.7)	
	**HMG**	4 (0.9)	5 (1.2)	
	**Progesterone prime**	1 (0.2)	0 (0)	0.21
Endometrial thickness (mm)**		9.02 ± 1.23 (2, 9)	8.95 ± 1.30 (1.8, 8.50)		0.13
*Data presented as Mean ± SD (IQR, MD), Student *t* test. **Data presented as Mean ± SD (IQR, MD), Mann-Whitney test. ***Data presented as n (%), Chi-square test. PRP: Platelet-rich plasma, BMI: Body mass index, COC: Cumulus-oocyte complex, IVF: In vitro fertilization, ICSI: Intracytoplasmic sperm injection, ART: Assisted reproductive technology, HMG: Human menopausal gonadotropin				

**Table 2:** Demographic characteristics of participants in 2 study groups


**Variables**	**PRP group (n = 217)**	**Control group (n = 101)**	**P-value**
Age (yr)*	31.03 ± 5.47 (7, 31)	31.96 ± 4.65 (8, 31)	0.21
BMI (kg/m^2^)*	26.21 ± 4.23 (4.8, 26.1)	26.41 ± 4.45 (5.6, 25.8)	0.69
Duration of infertility (year)*	7.38 ± 4.69 (5, 6)	6.69 ± 4.36 (5, 6)	0.22
COC**	20.21 ± 10.43 (15, 18)	20.91 ± 8.92 (14, 20)	0.28
Total number of embryos**	11.40 ± 6.56 (9, 10)	10.72 ± 0.59 (9, 10)	0.69
Number of transferred embryo**	1.94 ± 0.28 (0, 2)	1.95 ± 0.26 (0, 2)	0.64
Embryo quality***				
	**Good (A/B)**	205 (94.5)	96 (95)	
	**Bad (C/D)**	12 (5.5)	5 (5)	0.83
Type of fertilization***				
	**IVF**	18 (8.3)	8 (7.9)	
	**ICSI**	107 (49.3)	52 (51.5)	
	**IVF+ICSI**	92 (42.4)	41 (40.6)	0.93
ART cycle***				
	**Antagonist**	206 (94.9)	96 (95)		
	**Agonist**	3 (3.7)	3 (3)	
	**HMG**	2 (0.9)	2 (2)	
	**Progesterone prime**	1 (0.5)	0 (0)	0.75
Endometrial thickness (mm)*		9.09 ± 1.2	9.05 ± 1.4		0.82
*Data presented as Mean ± SD (IQR, MD), Student *t* test. **Data presented as Mean ± SD (IQR, MD), Mann-Whitney test. ***Data presented as n (%), Chi-square test. PRP: Platelet-rich plasma, BMI: Body mass index, COC: Cumulus-oocyte complex, IVF: In vitro fertilization, ICSI: Intracytoplasmic sperm injection, ART: Assisted reproductive technology, HMG: Human menopausal gonadotropin				

**Table 3:** Comparison of pregnancy outcomes between the 2 study groups


**Variables**	**PRP**	**Control**	**P-value**
**Chemical pregnancy**	128 (29.6)	89 (21.2)	< 0.001
**Clinical pregnancy**	104 (24.1)	58 (13.8)	< 0.001
**Ongoing pregnancy**	83 (19.2)	39 (9.3)	< 0.001
**Live birth**	82 (19)	39 (9.3)	< 0.001
**Abortion**	17/128 (13.2)	19/89 (21.3)	0.86
Data presented as n (%). Chi-square test. PRP: Platelet-rich plasma			

**Table 4:** Comparison of pregnancy outcomes between the 2 study groups considering implantation failure history


**Times of transfer**	**PRP**	**Control**	**P-value**
**First **	46	161	
**Chemical pregnancy**	14 (30.4)	28 (17.4)	0.08
**Clinical pregnancy**	9 (19.6)	18 (11.2)	0.17
**Ongoing pregnancy**	5 (10.9)	11 (6.8)	0.33
**Live birth**	5 (10.9)	11 (6.8)	0.27
**Second **	169	158	
**Chemical pregnancy**	42 (24.9)	40 (25.3)	0.97
**Clinical pregnancy**	37 (21.9)	26 (16.5)	0.20
**Ongoing pregnancy**	33 (19.5)	18 (11.4)	0.04
**Live birth**	32 (19.5)	18 (11.4)	0.04
**Third and more**	217	101	
**Chemical pregnancy**	72 (33.2)	21 (20.8)	0.03
**Clinical pregnancy**	58 (26.7)	14 (13.9)	0.01
**Ongoing pregnancy**	45 (20.7)	10 (9.9)	0.02
**Live birth**	44 (20.3)	10 (9.9)	0.02
Data presented as n (%). Chi-square test. PRP: Platelet-rich plasma			

**Table 5:** Comparison of pregnancy outcomes between the 2 study groups considering donor egg recipients


**Times of transfer**	**PRP**	**Control**	**P-value**
**First **	19	43	
**Chemical pregnancy**	5 (26.3)	8 (18.6)	0.49
**Clinical pregnancy**	3 (15.8)	4 (9.3)	0.45
**Ongoing pregnancy**	3 (15.8)	2 (4.7)	0.13
**Live birth**	3 (15.8)	2 (4.7)	0.13
**Second **	64	35	
**Chemical pregnancy**	15 (23.4)	8 (22.9)	0.94
**Clinical pregnancy**	13 (20.3)	7 (20)	0.97
**Ongoing pregnancy**	13 (20.3)	6 (17.1)	0.70
**Live birth**	13 (20.3)	6 (17.1)	0.70
**Third and more **	82	26	
**Chemical pregnancy**	30 (36.6)	4 (15.4)	0.09
**Clinical pregnancy**	21 (25.6)	2 (7.7)	0.05
**Ongoing pregnancy**	16 (19.5)	2 (7.7)	0.15
**Live birth**	16 (19.5)	2 (7.7)	0.15
Data presented as n (%). Chi-square test. PRP: Platelet-rich plasma			

## 4. Discussion

In this study, we assessed the pregnancy outcomes in 432 women who underwent intrauterine PRP treatment 48 hr before frozen-thawed embryo and compared it to a control group of 420. We found out that overall chemical, clinical, ongoing pregnancy, and live birth rates were higher in the PRP group, and the results were statistically significant.

In a more detailed analysis, it is determined that these parameters were only statistically significant if there was a history of at least 2 implantation failures present. Also, it was an interesting observation that although there was not a significantly higher pregnancy rate in women with a history of only one implantation failure, PRP treatment improved the likelihood of the live birth rate in this group.

In a 2023 randomized clinical trial, we compared the effect of PRP therapy on 33 women with a history of at least 2 prior implantation failures with the control group. We found that the PRP treatment before embryo transfer improved ART outcomes, that is, chemical, clinical, and ongoing pregnancy rates, but it was not statistically significant. It is worth mentioning that the size of our population study was small, and we did not evaluate the effect of the PRP treatment on the live birth rate and abortion rate (14).

However, in another 2018 RCT, we reported that PRP treatment in women with thin endometrium can significantly improve endometrial thickness alongside pregnancy rates (15). Similarly, Tehraninejad et al. reported similar results in a study of 85 women that PRP treatment does not seem effective in patients women with recurrent implantation failure (RIF) and normal endometrial thickness (7 mm) (16). Similar to our results, in a 2023 retrospective cohort study, the pregnancy outcomes of 64 women with RIF who received PRP before embryo transfer, compared with 54 in the control group and clinical, chemical, and live birth rates were significantly higher in the PRP group (17).

Also, a study in 2022 performed by Xu et al. on 288 women with RIF showed that the PRP therapy before embryo transfer could improve pregnancy outcomes (chemical and clinical pregnancy rates and live birth rate). They also mentioned that the PRP group had a higher implantation rate and a lower miscarriage rate, but it was not statistically significant (18).

In a 2023 systematic review and meta-analysis, the efficacy of the intrauterine infusion therapy, including granulocyte colony-stimulating factor, peripheral blood mononuclear cells, human chorionic gonadotropin, and PRP, in improving pregnancy outcomes in women with RIF were investigated, which included 21 studies with 2917 cases. It has been shown that the clinical pregnancy rate compared to the control groups was significantly higher in all 4 methods, although the live birth rate only improved in the PRP group. This is in line with our study results. Also, it has been demonstrated that PRP and PBMC had a higher ranking in improving the pregnancy rate and live birth rate. However, only G-CSF seemed to be effective in early abortion. In the end, it was concluded that all of these treatments can improve pregnancy outcomes, with the PRP being the most efficacious (19).

Deng et al. reported results in the same lane in their 2022 meta-analysis (20). On the other hand, a 2023 meta-analysis, recorded that there is little confidence that administration of the PRP had any significant effect on chemical and clinical pregnancy or live birth rates (21). This is in contrast to the Abd Elsalam Elgendy meta-analysis in 2023, which has reported improved chemical and clinical pregnancy rates as well as endometrial thickness in women with RIF after the PRP administration. It's worth mentioning that they did not assess its effect on the live birth rate (22).

According to all that mentioned above, it seems that whether the PRP could be a good approach to improve pregnancy outcomes in women with or without RIF needs more investigation and time to conclude.

Our results showed that in women who received donor eggs and had a history of repeated implantation failure, the PRP treatment also improves pregnancy outcomes as well. However, for live birth and ongoing pregnancy rates, it was not statistically significant which seems to be due to the small number of the control group (only 26). We could not find any study that would address this specific matter (intrauterine PRP treatment in women with RIF who used donor eggs). Considering that we can almost exclude the ovarian factor of infertility in this group, conducting more studies with sufficient sample sizes to establish results could be quite helpful in providing information about the effectiveness of the PRP in improving the chance of implantation.

## 5. Conclusion

Based on this study's results, it seems that PRP could be a beneficial approach in women with RIF and might be considered in patients with only one implantation failure to improve the live birth rate.

##  Data availability

The data that support the findings of this study are available from the corresponding author (Maryam Eftekhar), upon reasonable request.

##  Author contributions

Maryam Eftekhar and Neda Fatahi Meybodi designed the study and conducted the research. Monitoring, evaluating, and analyzing the data was done by all authors. Also all authors reviewed the article and approved the final manuscript and take responsibility for the integrity of the data.

##  Conflict of Interest

The authors declare that there is no conflict of interest.
